# Polyphenolic Nutraceuticals to Combat Oxidative Stress Through Microbiota Modulation

**DOI:** 10.3389/fphar.2019.00492

**Published:** 2019-05-03

**Authors:** Emanuel Vamanu

**Affiliations:** Faculty of Biotechnology, University of Agronomic Sciences and Veterinary Medicine of Bucharest, Bucharest, Romania

**Keywords:** biomarker, pattern, human, dysbiosis, antioxidant

## Abstract

Due to their direct relationship with the activity of the gut microbiota, nutraceuticals are, at present, an effective alternative for the mitigation and alleviation of the dysfunctions governed by oxidative stress. The escalation in the number of the target group patients (diabetes, cardiovascular dysfunction, cancer, etc.) has spurred the quest for alternative action methods. The therapeutic value is determined through *in vitro* and *in vivo* methods, and involves the analysis of the therapeutic index. As the adverse outcomes are decreased, the pharmacological potential is assessed by the mechanisms, including biotransformation and the identification of the relevant biomarkers. Inflammatory action is among the principal effects that need to be reduced because it favors the presence of free radicals and dysbiosis. This article aimed at highlighting the action of the nutraceuticals in minimizing the oxidative stress by directly influencing the microbiota and slowing down the inflammatory progression. The pharmacological aspects as a therapeutic indicator of the use of nutraceuticals in improving the population health.

## Introduction

Nutraceuticals have been suggested to alleviate oxidative stress-related diseases (Catinean et al., 2018). The term was recently defined as a compound that provides medical or health benefits, including the prevention and/or treatment of a disease (Santini et al., 2018). Although it is often the triggering cause, oxidative stress acts on a second plane, the real and primary cause being the disruption of the balance of the human colonic microbiota. By relating the oxidative stress with an inflammatory process, it was found that dysbiosis could be involved in disease development (Buttó and Haller, 2016). Recent studies have revealed the need to discover the biomarkers that can lead to the portrayal of the health status of those patients diagnosed with degenerative pathology (e.g., the cardiovascular patient group) (Hon et al., 2017). Human gut microbiota (all the microorganisms present in the human colon) is one such example, and the biotransformation of the nutraceuticals is a direct indicator of the presence of the biomarkers (e.g., butyric acid).

**FIGURE 1:**
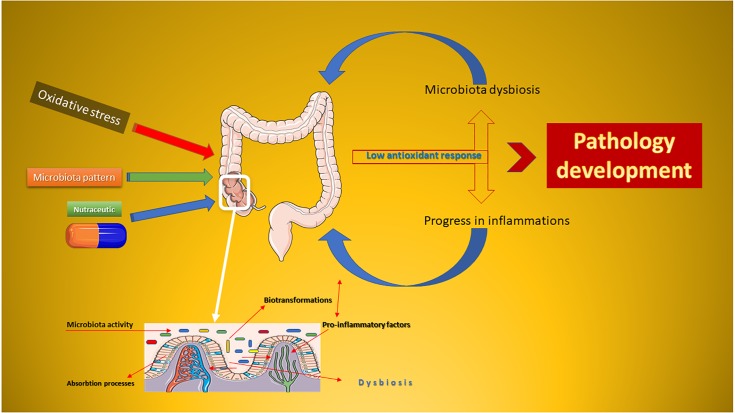
Schematic representation of the oxidative stress-dysbiosis-pathology progression. This Figure was obtained in part by using images from Servier Medical Art, licensed under CC-BY 3.0.CC BY 3.0.

Based on the evidence resulting from clinical practice, the microbiota is considered the second target for the drug and/or nutraceutical administration (Swanson, 2015). Compared to classical drugs, a nutraceutical drug is normally composed of a complex of bioactive substances, whose effect is affected by human colonic biotransformations (Marín et al., 2015). The metabolic syndrome progresses as a side effect of weight gain and metabolic alterations, and nutraceuticals are the ground-breaking strategy used to minimize chronic inflammation (Figure 1). The development of pathologies is closely associated with the imbalance of the microbiota of the human colon (Rani et al., 2016). In this mini-review, will be presented the potential of some polyphenolic nutraceuticals in reducing the oxidative stress by acting directly on the microbiota and combating the inflammatory progression. Pharmacological aspects will be also considered.

## Polyphenolic Compounds as Nutraceuticals

Nutraceuticals are principally sourced from food (fruits, vegetables, mushrooms) which delay oxidative progression, as demonstrated by several studies (Fawole and Opara, 2016). From the perspective of pharmaceutical importance, studies have revealed that the initial content of the biologically active compounds is not significant for the quantity of the phenols and antioxidant potential, due to gastrointestinal digestion (Olugbami et al., 2014). The implications from these studies are related to the protection against the proliferation of the tumor cells, immunomodulatory effect, and natural decrease in the inflammatory processes (Delgado-Adamez et al., 2014). From a pharmacodynamic perspective, when nutraceuticals interact with antibiotics the result is significant losses of the biological effect expressed in the human body (Chopra et al., 2010). While this does not imply pharmacotoxicity, there is a definite decrease in the inhibitory effect of the target strains due to declining bioavailability (Anadón et al., 2016). The importance of nutraceuticals lies in the amounts administered, particularly because of the complexity of the product to be used. At present, the objective of the studies is to facilitate the working of the nutraceuticals to raise the pharmacological activity of the active principle without toxicity (Vamanu and Vamanu, 2015; Baker, 2017).

Grapes have been identified to be a common source of nutraceuticals, particularly because of their high flavonoid content. The high flavonoid levels present in red grapes signify protection against cardiovascular progression (Caleja et al., 2017). Thus, the nutraceuticals in grapes are responsible for the antioxidant and anti-inflammatory effects, which provide cardio- and neuroprotection (Georgiev et al., 2014). In animals, the *in vivo* studies have revealed that the biological effect of the flavonoids in grapes is determined by their interaction with the colon microbiota. They suggest similar effects *in vitro*, even if different compounds are analyzed as chemical structures. They appear to be unabsorbed at this level and they selectively modulate the microbiota and health response (Cassidy and Minihane, 2016).

Another example is coffee, which in the current clinical practice is considered a cardioprotector, when consumed in moderation (Carlström and Larsson, 2018). Cardiovascular risk is diminished by the presence of polyphenolic compounds that reduce hypertension if absorbed in sufficient quantity (Miranda et al., 2016). Moderate coffee consumption also reduces the risk of type 2 diabetes (Pimentel et al., 2009). The pharmacological effect is due to the presence of chlorogenic acid and the lowering of the blood glucose is directly dependent on the quantity of this compound in the beverage consumed (Bidel and Tuomilehto, 2013).

Resveratrol, a nutraceutical often used to combat oxidative stress, exerts antioxidant and anti-inflammatory effects on the enzyme cyclooxygenase-1. It is present in the species of the berries of genus *Vaccinium* (Nasri et al., 2014), as well as in grapes. Stilbenes are also responsible for antitumour effects, assumed to be achieved through antioxidant protection (Rimando et al., 2004). A recent study has shown that resveratrol is degraded by the microbial constituents of the microbiota in dihydro-resveratrol (Kawabata et al., 2019). This results in a limitation or possible effect different from the parent compound. This phenomenon, common for polyphenolic components, is a step in the biotransformation process, which to some degree controls the response to oxidative stress.

An interesting example, with many direct clinical implications, is the use of curcumin, the main ingredient in turmeric (*Curcuma longa*). The pharmacological awareness arises from the antioxidant activity combined with a strong anti-inflammatory effect. This has attracted much attention because of its ability to pass through the blood-brain barrier and exhibit neuroprotective activity in neurodegenerative pathologies (Andrew and Izzo, 2017). The antioxidant effect is expressed as a protection against oxidative stress, which *in vivo* is observed as the oxidation of the lipid component (Mythri and Srinivas Bharath, 2012). The direct use has shown decreased bioavailability; however, curcumin is active on the human microbiota (Abdel-Lateef et al., 2016), where it also exerts noteworthy antimicrobial action. This is clearly seen against *Staphylococcus aureus, Salmonella* sp. and some *Candida* species (Singh et al., 2010; Pandit et al., 2015).

The *in vitro* effect of the curcumin on the HepG2 cancer cells was moderate; the curcumin directly administered affected the morpho-physiological aspect of the cells, but did not break down the cellular progression (Shoji et al., 2014). The methanolic extract was found to increase the bioavailability of the other phenolic components which had improved the effect of cellular multiplication (Tanvir et al., 2017). This mechanism was also dependent upon the effect of the modulation on the microbiota in cardiovascular patients (Vamanu and Sarbu, 2018), which demonstrated (*in vitro* simulation) a correction of the microbial pattern and a balance of the metabolic activity.

*In vitro*/*in vivo* results have shown that the pharmacological use of curcumin needs to be addressed from a low stability perspective, which affects bioavailability, pharmacokinetics and pharmacodynamics. Encapsulation with lipids may be an alternative to the delivery and action of a compound with limited stability. Microbiota is a target for the use of curcumin as a pharmaceutical ingredient because it can be a viable way to enhance limited absorption (Nelson et al., 2017).

The molecular pathways of caffeic acid to improve the glucose utilization continue to remain unclear. They are based on the glucose uptake in the adipocytes and support the insulin secretion. In this case, the nutraceuticals control the glucose levels by acting on the Na + / glucose co-transporter (SGLT1) at the intestinal level. The process determines the decrease in the glucose uptake at this level (Postal et al., 2014; Douglas, 2018).

## Cause-And-Effect of Microbiota Dysbiosis

One of the main causes of the occurrence of dysbiosis at a young age is excessive antibiotic intake. The widespread use of these pharmaceuticals as animal breeding factors has resulted in secondary administration, and the adverse effects have been countered via the use of pro-and prebiotics (Brugman et al., 2018). Although the initial effects were satisfactory, over time, the effectiveness of these products failed to correct the microbial fingerprint.

New bioactive molecules that are the target in the modern biopharmaceutical industry, like the polyphenol carboxylic acids, are active when they reach the colon. Although the absorption in the upper levels is poor, it directly affects the composition of the microbiota pattern in the colon (Espín et al., 2017). These compounds positively modify the microbiological ratio by favoring the *Lactobacillus* and *Bifidobacterium* strains (Vamanu et al., 2018). A prebiotic-like effect is demonstrated, which attenuates one of the principal causes of degenerative progression (Espín et al., 2017).

Biotransformation is determined by the fermentative action of the microbiota, and the employment of the nutraceuticals as the carbon source. This is one of the reasons for the inconclusive results obtained, for example, for pomegranate juice, (Williamson and Clifford, 2017). In such studies, the target is the anthocyanin content, because it offers protection against the inflammatory progression from oxidative stress (Mandal et al., 2017). After this juice is consumed, the anti-inflammatory process induces a decline in the various oxidative stress markers. The mechanism is meant to reduce the enzymes which encourage the proliferative process, as well as to protect against the decline in the nitric oxide level (Sohrab et al., 2014).

Chlorogenic acid, a compound present in several commonly used products (coffee), undergoes biotransformation in response to some favorable strains. Subsequently, the by-products (caffeic acid, for example) get absorbed. Thus, they exert a direct biological effect; the initial product has a side effect induced by the modulation of the microbial pattern. Although the direct *in vitro* effect is positive, the *in vivo* results are inconclusive, because the effect is expressed by the exclusive action of the products and the metabolism of the microbiota (Parkar et al., 2013). The presence of these compounds (in the feces and/or urine) is a biomarker of its utilization. It has been shown that several of the biotransformation products get eliminated in the urine, without averting the cause and with the excessive loss of the active substances (Gil-Sáncheza et al., 2018).

In clinical practice there is good reason to believe that dysbiosis is favored by diet, age, and genetic variability, and affects the progression of microbiome-linked diseases (Lozupone et al., 2012). The excess usage of antibiotics causes a mediated response that determines a microbial pattern change within a family having a well-established clinical history. The action mechanism transmitted through the interspecific relationships within the microbiota continues to remain a rather poorly explained fact. The evolution of degenerative diseases, particularly the neurodegenerative ones, looks to the host-microbiota interaction to produce new clinical approaches. Modulation of the dysbiosis passes the level of administration of certain strains that cannot develop interspecific linkages in the microbial pattern structure (Tremaroli and Bäckhed, 2012).

## Pharmacological Evidence of the Interactions of the Nutraceuticals/Drugs With the Human Microbiota

The pharmacodynamics of nutraceuticals differs from that of drugs, in that, the molecule complex possessing the bioactive potential in the first category does not act unidirectionally. The pharmacological effect is directly dependent upon the concentration of the main component, and therefore, different technologies are employed to potentiate the biological action (Vamanu, 2014). Metabolomic analysis is thus a tool that is effective in depicting the usefulness of some of the xenobiotic products (herbal extracts or active principles of drugs). This strategy determines, *in vivo*/*in vitro*, the ability of a product to express the effect with maximum efficiency, and to remove from use the specific products/compounds that exhibit antagonism (Lan et al., 2013).

Natural nutraceutical products (e.g., green tea) are a source of xenobiotics, already well-known for their effect against oxidative stress. A metabolomic study clearly depicts the active molecules and differences in their sources of origin, processing, and *in vivo* use. The interactions of the xenobiotics with human fluids (saliva, HCl, pepsin, bile salts, etc.), the biotransformations that occur, and the metabolite patterns determined by the action of the microbiota, affect the therapeutic effect. The interaction of the metabolomic pattern with the physiological functions will be reflected in the expression of the effect of health promotion (Fujimura et al., 2011). The principal aim of the investigation of the pharmacological action is to recognize the effect of the bacterial metabolites on the metabolism of the host, which is termed as ‘co-metabolism’ in the current studies. The discovery of the cause of the degenerative progression (obesity, diabetes, for instance) and the critical point of the installation of the dysbiosis, form an innovative method for improving the metabolic imbalance (Rajani and Jia, 2018).

Antibiotics are a drug class that interacts with the human microbiota. Their effect is directly dependent on the fermentative action of the colon, as they can be metabolized at this point. This implies being able to identify the metabolites after administration. This behavior resembles the effect of most products based on the phenolic compounds (nutraceuticals). Biological action is an expression of the presence of these metabolites and not of the biologically active molecules (Klaassen and Cui, 2015). Biotransformation in the colon controls the revision or reduction in the action of several of these *in vitro* products (Vamanu and Sarbu, 2018). The bioavailability rate at the individual level is determined by the modification of the inflammatory process, which during its evolution, changes the structure of the microbiota. The progressive increase in the oxidative stress, favoring the microbial modification, alters both the immune response and metabolic rate (Morgan et al., 2018). Thus, the microbiota is the principal factor in the treatments that target the absorption of the essential minerals (calcium), because it mediates the intestinal absorption and immune response related to the degradation of the bone mineral structure (Xu et al., 2017).

The bioavailability *in vivo* differs significantly from the findings of the *in vitro* studies. The therapeutic index is a useful parameter when investigating the pharmacological effect of a product/molecule (Lin et al., 2016). Interaction with the colonic microbiota is crucial for expressing the therapeutic value. While the high metabolic rate is specific to this category of bioactive molecules it is also a limiting factor. Reduced stability excludes the valuable compounds, which are ineffective *in vivo*, from the treatment schemes (Rein et al., 2013). The *in vivo* bioavailability revealed by the microbial metabolite products exerts a direct modifying effect on the patterns of different pathogenic groups. These components stimulate the multiplication of the favorable strains at the expense of the remainder of the microbial fingerprint. The clinical effect is thus indirectly exerted by the plasticity of the microbial pattern in the human colon. Any study on the interactions of the microbiota with the nutraceuticals involves not only mutual interaction, but also their clinical consequences on human health (Laparra and Sanz, 2010).

Interaction with the microbiota is expressed according to the type of biotransformation process that occurs, because the microbial pattern reacts and determines the clinical effect. Absorption is the direct effect of the degree of degradation and biotransformation that impacts the pharmacological response. Enzymatic action is the chief factor in the biotransformation process, through which the end-products are made stable and enabled to pass into the bloodstream. The shift from a water-soluble to liposoluble state is the distinctive means of raising the percentage of molecules absorbed in the intestinal lumen (Banks, 2009; Marín et al., 2015). This process is dependent upon the individual variability and the ability of the individual pattern to obtain those specific compounds (molecules) that can ensure a decrease in the microbial risk groups, for the evolution and stimulation of oxidative stress. The key to success not only in the administration of the polyphenols, but also in the reaction to the excess xenobiotics, is the escalation in the diversity of the intestinal microbiota. This complex process, influenced by several exogenous factors and individual genetic heritage, facilitates the biotransformation, absorption, and bioavailability of the nutraceuticals. Combination with dietary fiber and minerals supports the increase in bioavailability (Santini and Novellino, 2017).

Gram-positive bacteria, which include the pathogenic species as well (*Clostridium difficile, S. aureus*), directly affect the metabolisation of some drugs. This evidence confirms the part played by the entire microbiota in the pharmacological action of some drugs in the target groups (e.g., in treating cancer patients or obesity). For instance, doxorubicin, which induces cardiac damage, azidothymidine, which induces myopathy, or cisplatin, which induces ototoxicity, possess well-established mechanisms (Carmody and Turnbaugh, 2014). However, the induction of oxidative stress continues to remain a poorly understood biological phenomenon. The assumption is that the appearance and proliferation of the generation of free radicals depends upon the occurrence of dysbiosis. Negative strain proliferation indirectly causes the oxidative pressure to rise, decreases the antioxidant status, and causes the degenerative pathologies (Deavall et al., 2012).

A critical view of these data reiterated that the bioactivity of the nutraceuticals was dependent upon the chemical structure and stability during the time of the microbiota activity. The *in vitro* vs. *in vivo* trials revealed a second interesting perspective because it explains the physiological pathway of the degradation of the nutraceuticals (polyphenols) at the time of the fermentative action of the microbiota. A meta-analysis should be considered for each compound, because bioactivity represents a specific biological characteristic that enables a clearer understanding of the progression of the dysbiosis.

The role of nutraceuticals in the management of chronic diseases is controversial. Although they are thought to be useful at various stages of degenerative evolution, there is a misunderstanding of the mechanisms of action of nutraceuticals with a biopharmaceutical role (Daliu et al., 2019). Hence the need for a strict classification, based on the various interactions that nutraceuticals can have *in vivo*. Their use in disease management should also consider interacting with pharmaceuticals (Santini and Novellino, 2018). A nutraceutical should be understood, according to recent studies, as a compound or combination of compounds used to prevent or reduce the risk of disease. The pharmacological effect exerted by modulation of the microbiota as an endogenous factor (Santini and Novellino, 2017) makes it a proactive drug/product (Santini et al., 2017).

The nutraceuticals interact with human microbiota and affect the physiological balance of the body. This declines with age and the inflammatory proliferation which becomes more acute in the presence of oxidative stress. These critical points determine a decreased bioavailability, and finally dysbiosis.

Microbiological modulation of the microbiota and the metabolomic response depend on the capacity to colonize *in vivo*, which is a limiting factor for the *in vitro* study. Many studies have been conducted in static systems, and the dynamic response (similar to that of the *in vivo*) is assumed to be based on the identification of certain biomarkers (e.g., butyric acid level). The response is determined by a great number of factors, both exogenous and endogenous. The limiting potential arises from the low number of studies using target groups with a microbiota pattern which retains certain characteristics (e.g., high coliform levels in type 2 diabetes patients, Vamanu et al., 2018).

## Conclusion

In conclusion, drug administration like those of antibiotics or anti-inflammatory drugs, induces oxidative stress through the dynamics of a human-microbiota pattern. Individual variability reveals a specific response to the microbiota–drug interaction. When classical medication is combined with the nutraceuticals, it reduces the inflammatory pressure and preserves the effect of the microbial plasticity. Therefore, intervention against dysbiosis becomes the top priority for optimizing the anti-inflammatory response to the growing pressure of oxidative stress.

Nutraceuticals will be a way of promoting human wellbeing in the future, but the degree of valorisation will depend on the understanding of factors that regulate different physiological processes. The use of products (compounds) as part of a personalized treatment will be an essential point in increasing the quality of life (Zmora et al., 2018).

## Author Contributions

EV had a mini-review idea and wrote the manuscript.

## Conflict of Interest Statement

The author declares that the research was conducted in the absence of any commercial or financial relationships that could be construed as a potential conflict of interest.
